# Discovery and biochemical characterization of the D-aspartyl endopeptidase activity of the serine protease LACTB

**DOI:** 10.1016/j.jbc.2025.108549

**Published:** 2025-04-24

**Authors:** Genta Ito, Naoko Utsunomiya-Tate

**Affiliations:** Department of Biomolecular Chemistry, Faculty of Pharmaceutical Sciences, Teikyo University, Tokyo, Japan

**Keywords:** D-aspartyl endopeptidase, DAEP, LACTB, D-aspartate, isomerization, racemization, AlphaFold, Dali

## Abstract

Nonenzymatic D-isomerization of aspartic acid in proteins has been observed in lesions associated with age-related diseases, including cataracts and Alzheimer's disease. Given that D-isomerization of Asp disrupts the physiological conformation of proteins, it has been postulated that D-isomerization of Asp in proteins is a key factor in the pathogenesis of age-related diseases. D-Aspartyl endopeptidase (DAEP) activity, which cleaves proteins at the carboxy terminus of D-Asp and potentially induces degradation of abnormal proteins with D-isomerized Asp, has been observed in mitochondrial fractions of mammalian tissues. However, the specific proteins responsible for mammalian DAEP activity remain unknown. In this study, we identified mitochondrial serine β-lactamase–like protein (LACTB) as the first mammalian protein with DAEP activity by structural comparison with paenidase, a bacterial DAEP. LACTB exhibited DAEP activity similar to paenidase in an *in vitro* assay. In addition, LACTB cleaved a 10-residue peptide derived from amyloid β1–10 containing D-Asp at position 7, which was also observed with mammalian DAEP. LACTB has previously been characterized as a tumor suppressor and as a protein whose increased expression is associated with an increased risk of Alzheimer's disease. Therefore, our findings suggest that disruption of the proteostasis of D-Asp–containing proteins may underlie the pathogenesis of these diseases.

Amino acid residues in proteins are exclusively in the L-form immediately after ribosomal translation. However, increasing evidence suggests that a subset of Asp residues undergo isomerization to the D-form *via* a nonenzymatic process in long-lived proteins and when proteostasis is compromised, such as in the context of aging (1). Such D-isomerization of Asp has been observed in various proteins, including amyloid β (Aβ) peptides that accumulate in the brains of patients with Alzheimer's disease (2, 3) and crystallin proteins in aged eyes (4). Consequently, D-isomerization of Asp in proteins has been postulated to disrupt their native 3D structure, leading to dysfunction and/or degeneration and contributing to the pathogenesis of age-related diseases. We have shown previously that site-specific D-isomerization of Asp in Aβ and tau, which also accumulates in the brains of Alzheimer's disease patients, has differential effects on their β-sheet transition and fibril formation *in vitro* depending on the Asp residues isomerized (5, 6, 7).

Given the deleterious effects that D-isomerization of Asp has on protein structure and function, organisms have evolved several defense mechanisms to overcome this isomerization. For example, mammalian cells possess protein isoaspartyl (D-aspartyl) *O*-methyltransferase, an enzyme that reverts isomerized Asp back to L-Asp (8). Another mechanism is the existence of proteins with D-aspartyl endopeptidase (DAEP) activity, which cleave peptides at the carboxy terminus of D-isomerized Asp, leading to degradation. Although DAEP activity has been found in mitochondrial fractions from rabbit tissues (9), the protein(s) responsible for DAEP activity remains unresolved.

The only protein shown to have DAEP activity is paenidase (PAE), which was found in the culture medium of Paenibacillus bacteria (10, 11). Both PAE and mammalian DAEP have been observed to have similar activities, including endopeptidase activity against a 10-residue peptide derived from Aβ (Aβ1–10; NH_2_-DAEFRHDSGY-COOH), cleaving this peptide at the carboxy terminus of Asp7 only when D-Asp is present at position 7 (*i.e.*, NH_2_-DAEFRH [D-Asp] SGY-COOH) (9, 10). Both PAE and mammalian DAEP are inhibited by i-DAEP, a small molecule developed by modifying Aβ1–10 [D-Asp7] (9, 10).

In this study, to identify proteins responsible for mammalian DAEP activity, we searched for human proteins with structures similar to PAE using AlphaFold2 (AF2) and the Dali server and found that mitochondrial serine beta-lactamase-like protein (LACTB) is structurally similar to PAE and exhibits DAEP activity *in vitro*.

## Results

### Structure prediction of PAE using AF2 and RoseTTAFold

**Figure 1:**
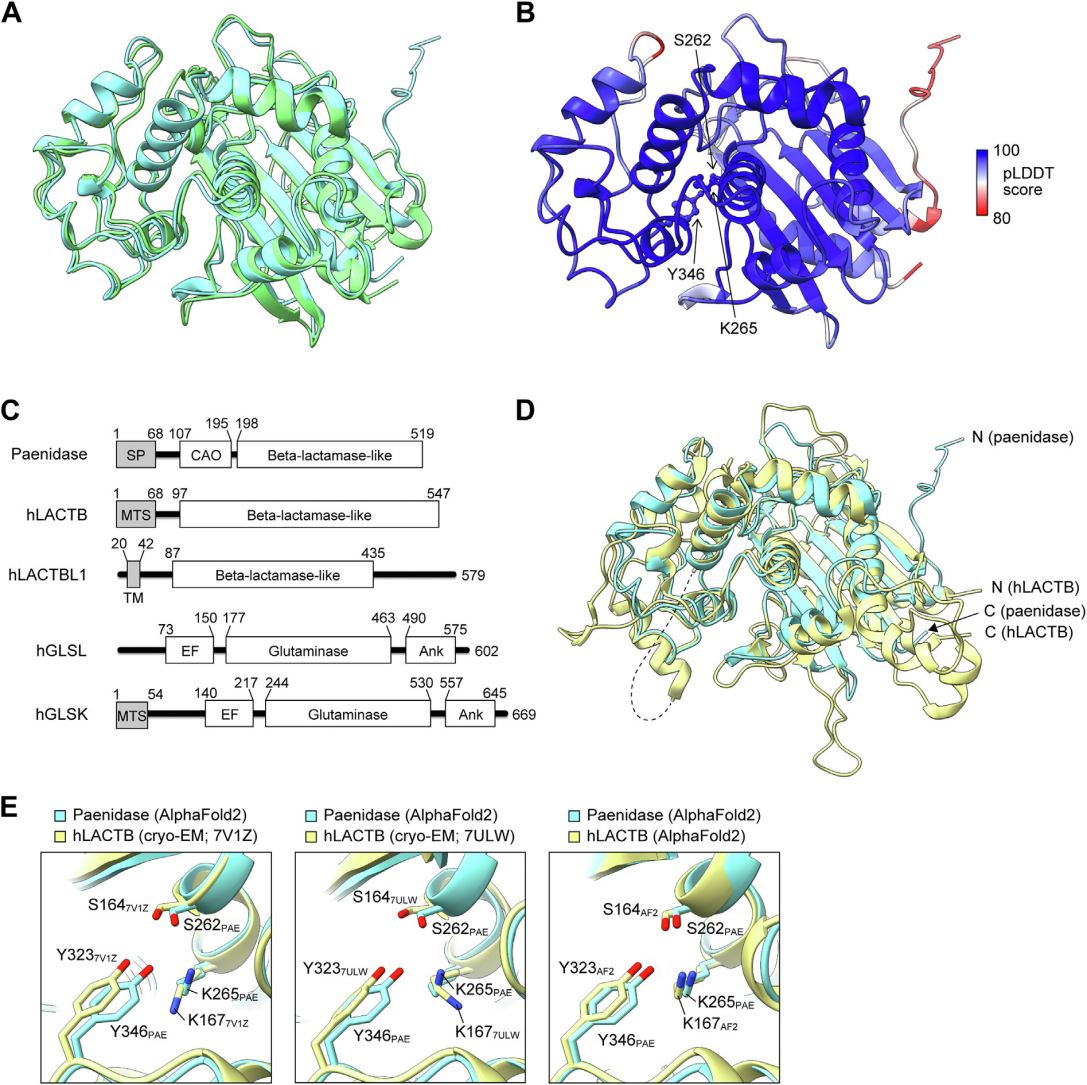
**Identification of human proteins structurally similar to paenidase.***A*, superposition of paenidase (PAE) structures predicted by AlphaFold2 (AF2) (*cyan*) and RoseTTAFold (*light green*). *B*, color-coded AF2 structure of PAE based on pLDDT scores. Three catalytic residues are indicated by *arrows*. *C*, domain architectures of PAE and human proteins identified as structurally similar to PAE. *D*, superposition of the structures of PAE predicted by AF2 (*cyan*) and human LACTB (hLACTB) determined by cryo-EM (PDB ID: 7V1Z, chain C; *light yellow*). *E*, close-up of the superimposed putative catalytic center of hLACTB (*light yellow*) and PAE (*cyan*). The AF2 structure of PAE is superimposed on one of two structures of hLACTB determined by cryo-EM (PDB ID: 7V1Z (*left*) or 7ULW (*middle*)) or on the AF2 structure of hLACTB (*right*). Side chains of putative catalytic residues are shown, with oxygen and nitrogen atoms marked in *red* and *blue*, respectively. Ank, ankyrin repeats; CAO, Cu-containing amine oxidase domain; EF, EF-hand domain; LACTB, serine beta-lactamase-like protein; MTS, mitochondrial targeting signal; pLDDT, predicted local distance difference test; SP, signal peptide; TM, transmembrane domain.

The 3D structure of mature PAE (198–519 aa) was predicted using AF2 (12) and RoseTTAFold (13). The two prediction algorithms yielded a similar protein structure consisting of a beta-lactamase structural motif containing a cluster of α-helices and an α/β sandwich (Fig. 1*A*). The predicted local distance difference test scores calculated by AF2 were above 90 in most of the core region (Fig. 1*B*), suggesting that the predicted core structure is highly reliable. A structural comparison of the two predicted structures was performed using the Dali server, which yielded a Z-score of 45.2 and a RMSD of 1.4 Å between the aligned 312 Cα atoms out of a total of 322 Cα atoms in a 3D superposition. These values indicate that the two predicted structures were considerably similar.

### Identification of human proteins with structures similar to PAE

The Dali server was used to search the AF2 database to identify human proteins with 3D structural similarities to the mature PAE model. The top hit was LACTB with a Dali Z-score of 38.0 and a Cα RMSD of 2.4 Å (Table 1). The second top hit was putative beta-lactamase–like 1 (LACTBL1) with a Dali Z-score of 35.2 and a Cα RMSD of 2.4 Å (Table 1). These results indicate that the structures of LACTB and LACTBL1 predicted by AF2 are similar to the predicted structure of mature PAE. There were also two glutaminases, a liver isoform (GLSL) and a kidney isoform (GLSK), whose predicted structures showed moderate similarity to the mature PAE model (Table 1). Domain prediction using the InterPro server (14) revealed that GLSL and GLSK contain the glutaminase fold (InterPro: 15868), which is a member of the beta-lactamase-like fold superfamily (InterPro: 12338). These results indicate that all four proteins have a beta-lactamase-like fold in their catalytic domains (Fig. 1*C*).

**Table 1:** Search results using the Dali server for human proteins with structures similar to paenidase

No.	Chain	Z-score	RMSD (Å)	LALI	# Residues	Identity (%)	PDB description	Protein name
1	fdh7-A	38.0	2.4	316	547	24	HUMAN:AF-P83111-F1	Serine beta-lactamase–like protein LACTB, mitochondrial
2	fgrh-A	35.2	2.4	301	500	24	HUMAN:AF-A8MY62-F1	Putative beta-lactamase–like 1
3	e8kf-A	12.6	5.0	214	602	12	HUMAN:AF-Q9UI32-F1	Glutaminase liver isoform, mitochondrial
4	fafx-A	10.8	5.1	215	669	13	HUMAN:AF-O94925-F1	Glutaminase kidney isoform, mitochondrial

Proteins with a Z-score greater than 10 are shown. “Chain” is a unique identifier in Dali. The “Z-score” indicates how similar the two structures are. Z-scores greater than 20 mean that the two structures are definitely homologous (34). “RMSD” is the root-mean-square deviation of the distance between aligned Cα atoms in a three-dimensional superposition. The number of paenidase equivalent residues is shown in the “LALI” column, whereas the total number of residues is shown in the “# Residues” column.

LACTB, serine beta-lactamase-like protein.

The 3D structure of LACTB has already been determined experimentally by cryo-EM (15, 16). The LACTB structure predicted by AF2 agreed well with the cryo-EM structure, with a Dali Z-score of 58.0 and a Cα RMSD of 1.0 Å, demonstrating the accuracy of the AF2 structure prediction. Comparison of the mature PAE structure predicted by AF2 with the cryo-EM structure of LACTB (PDB ID: 7V1Z, (15)) revealed a Dali Z-score of 35.5 and a Cα RMSD of 1.8 Å (Fig. 1*D*). Although there is an indeterminate region in the cryo-EM structure of LACTB (*i.e.*, 227–297 aa of LACTB, dashed line in Fig. 1*D*; underlined in Fig. S1), the overall structures are quite similar, as predicted by the comparison between the AF2 structures.

Two catalytic residues in PAE, namely Ser262 and Lys265, are within the SXXK motif conserved among the beta-lactamase superfamily, and the four hit proteins also possess the SXXK motif (Fig. S1). Tyr346 is another residue required for PAE DAEP activity and is conserved only in LACTB and LACTBL1 (Fig. S1). The side chains of Ser164 and Tyr323 in LACTB are oriented similarly to those in PAE (Fig. 1*E*). In contrast, the amino group of Lys167 in LACTB (K167_7V1Z_) is oriented in the opposite direction to the corresponding Lys265 in PAE (K265_PAE_) (Fig. 1*E*, left), which was also the case in another cryo-EM structure of LACTB (PDB ID: 7ULW (16); K167_7ULW_ in Fig. 1*E*, middle). Conversely, the side chain conformation of Lys167 in the AF2 structure of LACTB (K167_AF2_) was similar to that of K265_PAE_ (Fig. 1*E*, right).

### Evaluation of DAEP activity of PAE-like proteins

The DAEP activity of the four hit proteins was investigated by expressing their putative catalytic domains in *Escherichia coli* as amino-terminal 6His-SUMO1 fusion proteins. Although 6His-SUMO1-human LACTBL1 (hLACTBL1) (63–450) was insoluble in *E. coli* (Fig. S2*D*), which prevented purification, we were able to semipurify 6His-SUMO1-hLACTB (97–547) (Fig. S2*A*), 6His-SUMO1-hGLSL (148–478) (Fig. S2*E*), and 6His-SUMO1-hGLSK (221–533) (Fig. S2*H*) using Ni^2+^-nitriloacetic acid agarose columns. We then used size-exclusion chromatography to further purify 6His-SUMO1-hLACTB (97–547) (Fig. S2, *B* and *C*), hGLSL (148–478) (Fig. S2, *F* and *G*), and hGLSK (221–533) (Fig. S2, *I* and *J*) to near homogeneity.

**Figure 2:**
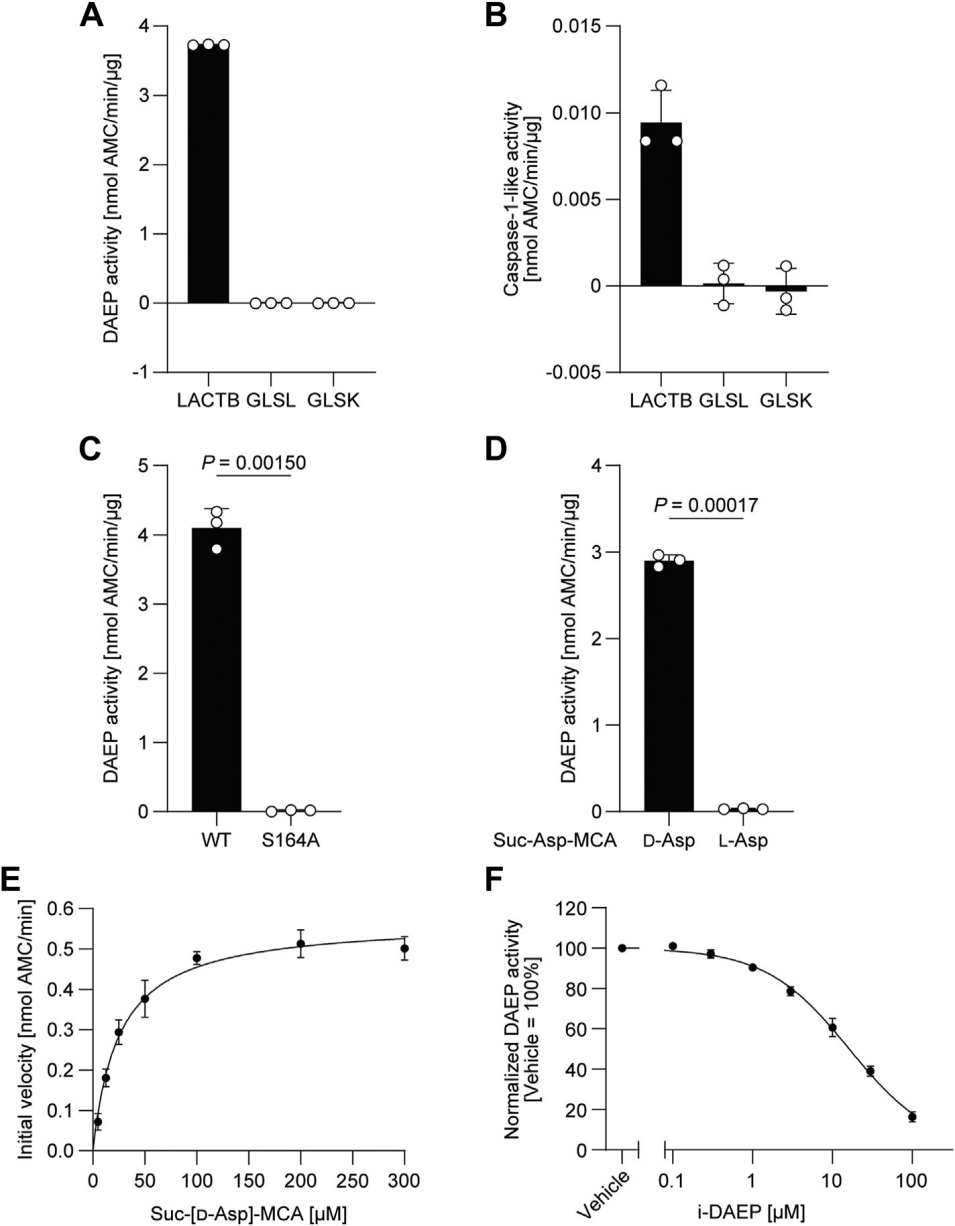
**DAEP activity of LACTB and inhibition by i-DAEP.** DAEP activity (*A*) and caspase-1–like activity (*B*) of LACTB, GLSL and GLSK assayed *in vitro*. *C*, DAEP activity of hLACTB WT and the S164A mutant. *D*, DAEP activity of hLACTB WT on Suc-[D-Asp]-MCA or Suc-[L-Asp]-MCA. In (*A–D*), *white circles* represent values from three independent experiments, each performed in triplicate, and *black bars* and error bars are the mean and SD, respectively. *E*, determination of the *K*_m_ value of LACTB DAEP activity on Suc-[D-Asp]-MCA. *F*, LACTB DAEP activity in the presence of various concentrations of i-DAEP. The Suc-[D-Asp]-MCA concentration was kept at 20 μM. The initial reaction rates were calculated and normalized by the rate of the vehicle control. In (*E* and *F*), *black circles* and error bars are the mean and SD, respectively, of three independent experiments, each performed in triplicate. Details of the statistical analyses are reported in Table S4. DAEP, D-Aspartyl endopeptidase; LACTB, serine beta-lactamase-like protein; Suc-[D-Asp]-MCA, succinyl-D-aspartic acid α-(4-methylcoumaryl-7-amide).

*In vitro* DAEP activity was measured using succinyl-D-aspartic acid α-(4-methylcoumaryl-7-amide) (Suc-[D-Asp]-MCA) as an artificial substrate. Nonfluorescent Suc-[D-Asp]-MCA is cleaved at the carboxy terminus of D-Asp by DAEP activity to release the MCA moiety as 7-amino-4-methylcoumarin (AMC), which becomes fluorescent. By quantifying AMC fluorescence, we showed that 6His-SUMO1-hLACTB (97–547) WT (hLACTB WT) exhibited DAEP activity, whereas 6His-SUMO1-hGLSL (148–478) and 6His-SUMO1-hGLSK (221–533) did not (Fig. 2*A*). As LACTB has been reported to have endopeptidase activity toward the caspase-1 substrate (*i.e.*, acetyl-L-tyrosyl-L-valyl-L-alanyl-L-aspartic acid α-(4-methylcoumaryl-7-amide) [Ac-YVAD-MCA]) (17), we also examined the caspase-1–like activity of the purified proteins. hLACTB WT showed caspase-1–like activity; however, this activity was very low compared with the observed DAEP activity (Fig. 2*B*).

We also expressed and purified the S164A mutant form of 6His-SUMO1-hLACTB (97–547) (hLACTB S164A), in which the putative catalytic serine in the SXXK motif was replaced by alanine to produce a mutant lacking DAEP activity. The DAEP activity of hLACTB S164A was negligible (Fig. 2*C*), indicating that Ser164 of LACTB functions as a catalytic residue, as in PAE. This result also showed that the DAEP activity of purified hLACTB WT is because of the LACTB protein and not because of other proteins contaminating the purified fractions. We also systematically examined the proteins present in the purified fractions of hLACTB WT and S164A by mass spectrometry and did not find any proteins that may explain the difference in DAEP activity between WT and S164A (Table S5). The DAEP activity of hLACTB WT on Suc-[L-Asp]-MCA was significantly lower than that on Suc-[D-Asp]-MCA (Fig. 2*D*), indicating the selectivity of LACTB DAEP activity toward D-Asp–containing substrates. The Michaelis constant (*K*_m_) of hLACTB WT for Suc-[D-Asp]-MCA was 25.3 ± 2.7 μM, and *k*_cat_ and *k*_cat_/*K*_m_ were 6.02 ± 0.12 s^−1^ and 0.24 ± 0.03 s^−1^ μM^−1^, respectively (Fig. 2*E*).

Mammalian DAEP was shown to be inhibited by i-DAEP with an IC_50_ value of 3 μM (9). hLACTB WT was also inhibited by i-DAEP, although the IC_50_ value of 13.6 ± 1.4 μM was less potent compared with the inhibition of mammalian DAEP (Fig. 2*F*).

### Cleavage of A**β**1–10 [D-Asp7] peptide by LACTB

**Figure 3:**
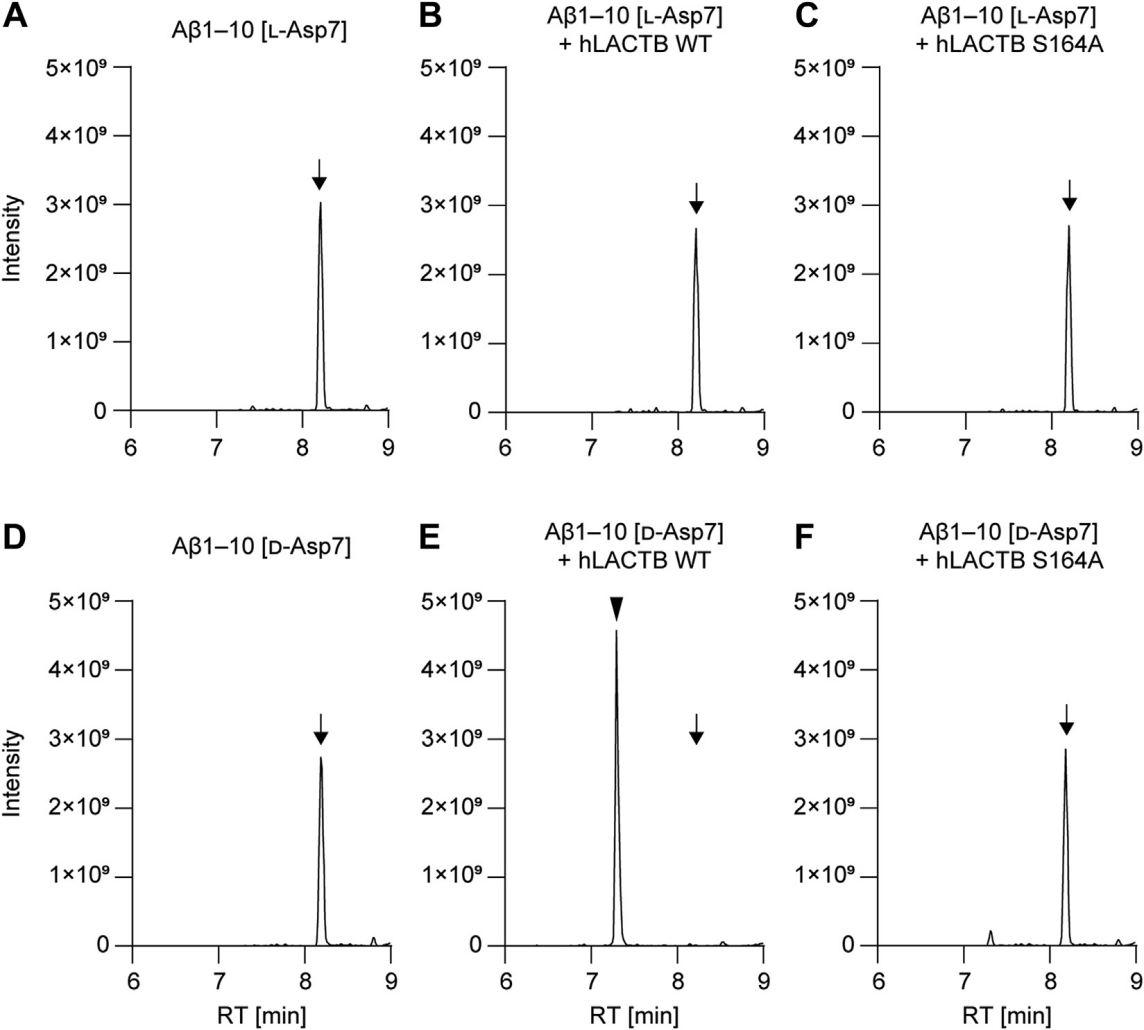
**Cleavage of Aβ1–10 [D-Asp7] peptide by LACTB.** Peaks of Aβ1–10 (*arrows*) and Aβ1–7 (*arrowheads*) in the base peak chromatograms obtained in LC-MS/MS analyses are shown. Aβ1–10 [L-Asp7] (*A–C*) or Aβ1–10 [D-Asp7] (*D–F*) was used as the substrate. hLACTB WT (*B* and *E*) or the S164A mutant (*C* and *F*) was used as enzyme, whereas storage buffer was used as the negative control in (*A* and *D*). Aβ, amyloid β; LACTB, beta-lactamase.

Mammalian DAEP has been shown to cleave the Aβ1–10 peptide (*i.e.*, NH_2_-DAEFRHDSGY-COOH) into Aβ1–7 and Aβ8–10 when the peptide contains a D-Asp at position 7 (Aβ1–10 [D-Asp7]) (9). Thus, the possible cleavage of Aβ1–10 [D-Asp7] by LACTB was investigated by incubating hLACTB WT or S164A with the Aβ1–10 peptide for 4 h at 30 °C, and the end products were analyzed by LC-MS/MS. In the base peak chromatograms, the peak corresponding to uncleaved Aβ1–10 was observed at 8.20 to 8.22 min in samples containing only Aβ1–10 [L-Asp7] or Aβ1–10 [D-Asp7] (arrows in Fig. 3, *A* and *D*). When hLACTB WT was incubated with Aβ1–10 [D-Asp7], a new peak was observed at 7.30 min (an arrowhead in Fig. 3*E*), and the Aβ1–10 peak disappeared (an arrow in Fig. 3*E*), indicating that the Aβ1–10 [D-Asp7] peptide was cleaved by hLACTB WT and that the new peak corresponded to the cleavage products. The new peak was not observed when Aβ1–10 [L-Asp7] was incubated with hLACTB WT (Fig. 3*B*), indicating the specificity for Aβ1–10 [D-Asp7] over Aβ1–10 [L-Asp7]. Samples of Aβ1–10 [L-Asp7] or Aβ1–10 [D-Asp7] incubated with hLACTB S164A did not show the cleavage product peak in the base peak chromatograms (Fig. 3, *C* and *F*), demonstrating that cleavage of Aβ1–10 [D-Asp7] by hLACTB is Ser164-dependent.

Extracted ion chromatograms (XICs) of ions corresponding to Aβ1–10 (Fig. S3*A*; *m/z* = 598.7459–598.7579; charge: +2) and Aβ1–7 (Fig. S3*B*; *m/z* = 445.1890–445.1980; charge: +2) showed that the peaks at 8.20 min and 7.30 min correspond to Aβ1–10 and Aβ1–7, respectively. In addition, an XIC of an ion corresponding to Aβ8–10 (*m/z* = 326.1313–326.1379; charge: +1) showed a peak at 7.35 min only when Aβ1–10 [D-Asp7] was incubated with hLACTB WT (Fig. S3*C*). These cleavage product peaks (*i.e.* Aβ1–7 and Aβ8–10) were significantly reduced when Aβ1–10 [D-Asp7] was incubated with hLACTB S164A (Fig. S3, *B* and *C*). The amino acid sequences of these peaks were also verified by tandem mass spectrometry (Fig. S4). These results indicate that hLACTB cleaves Aβ1–10 into Aβ1–7 and Aβ8–10 when it contains D-Asp at position 7.

## Discussion

Mammalian tissue homogenates have been shown to exhibit DAEP activity, suggesting that proteins with DAEP activity exist in mammalian tissues (9). In this study, we found that LACTB has DAEP activity *in vitro*. LACTB has been reported to be a tumor suppressor (17), and decreased expression of LACTB has been observed in various cancers, including breast cancer (17), colon cancer (18), glioma (19), ovarian cancer (20) and liver cancer (21). Reduced LACTB expression has also been shown to be associated with poor overall survival in these cancer patients (18, 19, 20, 21), although the underlying mechanisms remain unknown. Conversely, increased LACTB expression levels have been reported to be associated with increased risk of Alzheimer's disease in integrative analyses of genome-wide, proteome-wide, and transcriptome-wide association studies (22, 23). These observations suggest that alterations in the DAEP activity of LACTB may cause the onset and progression of these diseases.

LACTB has been characterized as a serine protease whose activity was assayed using Ac-YVAD-MCA, an artificial caspase-1 substrate (17). In this study, we demonstrated that the activity of LACTB against Suc-[D-Asp]-MCA is more potent than that against Ac-YVAD-MCA (Fig. 2, *A* and *B*). Detailed enzymological parameters of LACTB on Ac-YVAD-MCA were not available in previous literature, but the *V*_max_ on Ac-YVAD-MCA was approximately 5 to 10 pmol/min in the reaction scale identical to ours (15, 17), whereas that on Suc-[D-Asp]-MCA was 0.5 nmol/min in this study. The *k*_cat_ and *k*_cat_/*K*_m_ of LACTB on Suc-[D-Asp]-MCA were 6.02 s^−1^ and 0.24 s^−1^ μM^−1^, respectively, which were within the standard range for an endopeptidase (24, 25, 26). These results suggest that peptides containing D-Asp may be the *in vivo* substrate of the serine protease activity of LACTB.

The structural basis for the D-Asp selectivity of DAEP remains unknown. In the AF2 structure of PAE, the side chain of K265_PAE_ was oriented in the opposite direction to that of K167_7V1Z_ and K167_7ULW_ in the two cryo-EM structures of hLACTB but was oriented in the same direction as K165_AF2_ (Fig. 1*E*). The predicted local distance difference test score for K265_PAE_ is 98.8, indicating a high degree of structural confidence. On the other hand, the B-factors for K167_7V1Z_ and K167_7ULW_ are 45 and 122 Å^2^, respectively, indicating that the conformation of K167 contains relatively large fluctuations in these structures. Therefore, it is possible that the orientation of the Lys167 side chain is incorrectly modeled in the cryo-EM structures of hLACTB. Alternatively, it is possible that the orientation of the Lys side chain is what distinguishes DAEP-type from non-DAEP-type beta-lactamase-like proteins. If the AF2 structure of PAE is based on the experimental structure of beta-lactamase-like proteins without DAEP activity (*e.g.*, D-Ala-D-Ala carboxypeptidase and class C β-lactamase; Fig. S5), the AF2 structure of PAE may be biased toward non-DAEP-type proteins. It will be important to study the structures of PAE and LACTB in complex with competitive inhibitors such as i-DAEP or a peptide substrate such as Aβ1–10 to find the structural determinant of DAEP activity.

We used Aβ1–10 as an artificial peptide substrate for LACTB, although it does not seem to be relevant for the pathogenesis of Alzheimer's disease as Aβ is secreted into the extracellular space, whereas LACTB is located in the mitochondrial intermembrane space (IMS). Interestingly, LACTB has been reported to form a filamentous structure in the IMS (27), which is required for its caspase-1–like serine protease activity (15). As LACTB is conserved from *Caenorhabditis elegans* to *Homo sapiens*, it will be important to investigate the biological and pathological significance of its DAEP activity and filament formation by searching for proteins physiologically cleaved by LACTB in the IMS.

In conclusion, we have identified LACTB for the first time as a eukaryotic protein with *in vitro* DAEP activity. Further investigation of whether degradation of proteins containing D-isomerized Asp by LACTB occurs physiologically and serves as a defense mechanism against protein racemization in eukaryotic cells, especially in mitochondria, or whether site-specific cleavage of proteins containing D-isomerized Asp plays a key role in physiologically relevant signaling pathways will elucidate the importance of currently unstudied biological and pathological roles of D-isomerized Asp in proteins.

## Experimental procedures

### Bioinformatics: structure prediction and comparison

Structure prediction was performed using AF2 (12, 28) using MMseqs2 *via* ColabFold v1.5.5 (29) and/or RoseTTAFold (13) *via* the Robetta server (https://robetta.bakerlab.org/, last accessed Feb 3, 2025). For both programs, the amino acid sequence of mature PAE (*i.e.*, 198–519 aa) (UniProt ID: A0A2Z6BCG6_9BACL) was used as input and default parameters were used. For AF2, the rank 1 structure model was selected for further analysis from the five PDB files generated. For RoseTTAFold, model 1 was selected for further analysis from the five generated PDB files.

The generated PDB files were uploaded to the Dali server (30) (http://ekhidna2.biocenter.helsinki.fi/dali/, last accessed on Feb 3, 2025) to search for human proteins with structural similarity to the predicted structure of mature PAE. For this purpose, the “AF-DB comparison” mode was used. A structural comparison of two proteins was also performed using the Dali server by uploading two PDB files to the server. In this case, the “pairwise” mode was used. The protein structures were visualized and analyzed using UCSF ChimeraX (31). Amino acid sequence alignment was performed using the Clustal Omega web server (https://www.ebi.ac.uk/jdispatcher/msa/clustalo, last accessed Feb 3, 2025). Domain prediction was performed using the InterPro web server (https://www.ebi.ac.uk/interpro/, last accessed Feb 3, 2025).

### Reagents

(Suc-[D-Asp]-MCA), Suc-[L-Asp]-MCA, Ac-YVAD-MCA, AMC, and (benzoyl-L-arginyl-L-histidyl-D-aspart-1-yl) chloromethane (i-DAEP) were purchased from Peptide Institute Inc and dissolved in dimethyl sulfoxide. Peptides corresponding to amino acids 1 to 10 of human Aβ (Aβ1–10) with L-Asp7 or D-Asp7 were custom synthesized by Scrum Inc., Japan.

### Protein purification and *in vitro* DAEP assay using fluorescent substrates

Plasmids for bacterial expression encoding N-terminal 6His-SUMO1-fusion proteins were constructed as described in the Supporting Information. Protein expression and purification was performed essentially as described previously (32) and detailed methods are also provided in the Supporting Information.

For endpoint DAEP assays, 100 μl reaction mixtures (50 mM Tris–HCl, 150 mM NaCl, 200 μM Suc-[D-Asp]-MCA, 100 ng purified proteins, pH 8.0) were prepared in triplicate in 1.5 ml tubes. Reactions were initiated by adding 6His-SUMO1-fusion proteins and incubating at 30 °C with shaking at 1000 rpm for 5 min. The reaction was terminated by adding 900 μl 10% (v/v) acetic acid. For determining caspase-1–like activity, 500 μM Ac-YVAD-MCA was used as the substrate instead of Suc-[D-Asp]-MCA. The fluorescence of AMC generated by DAEP activity was measured using a spectrofluorometer (FP8300, Jasco). The measurement parameters were as follows: excitation wavelength: 380 nm; fluorescence wavelength: 460 nm; bandwidth: 5 nm; response: 1 s; sensitivity: 300 V; integration: 4 times. The results of three independent assays, each performed in triplicate, were used to calculate specific activity.

For kinetic DAEP assays, 50 μl of LACTB solution (2 ng/μl 6His-SUMO1-LACTB (97–547), 20 mM Tris–HCl, 150 mM NaCl, 1 mM DTT, 10% (v/v) glycerol, pH 8.0) were aliquoted into 96-well black plates and prewarmed for 5 min at 30 °C in a plate reader (SpectraMax i3x, Molecular Device), and 50 μl of substrate mixtures (50 mM Tris–HCl, 150 mM NaCl, 400 μM Suc-[D-Asp]-MCA, pH 8.0) were added to LACTB to initiate reactions. The plate was incubated at 30 °C and shaken at 1000 rpm for 5 s before each measurement. AMC fluorescence was monitored every 30 s for 5 min, and the first three measurements were used to calculate the initial reaction rate. The results of three independent measurements, each performed in triplicate, were used to calculate the kinetic parameters.

### Detection of the endoproteolytic activity toward A**β**1–10 peptides by LC-MS/MS

Reaction mixtures were prepared in Protein LoBind 1.5 ml tubes (Eppendorf) in 100 μl (50 mM Tris–HCl, pH 8.0, 50 μM Aβ1–10, 0.4 μg 6His-SUMO1-hLACTB (97–547)) and incubated at 30 °C for 4 h with shaking at 1000 rpm. The reactions were stopped by adding formic acid at a final concentration of 0.1% (v/v). The samples were filtered through 0.2 μm hydrophilic polytetrafluoroethylene spin filters (Pall Corporation) and diluted 100-fold with 0.1% (v/v) formic acid. One microliter of the diluted samples was analyzed by nano LC-MS/MS on Q Exactive coupled with Vanquish Neo (Thermo Fisher Scientific). The parameters for analysis are shown in Table S3. Base peak chromatograms and XICs were generated using Qual Browser (Thermo Fisher Scientific), and exported values were processed using GraphPad Prism 8 (GraphPad Software; https://www.graphpad.com/). The mass ranges used to generate XICs were as follows: Aβ1–10, *m/z* = 598.7459 to 598.7579 (+2 charge); Aβ1–7, *m/z* = 445.1890 to 445.1980 (+2 charge); Aβ8–10, *m/z* = 326.1313 to 326.1379 (+1 charge). Raw mass spectrometry data were processed using FragPipe v22.0 (33), and MS2 spectra of each peptide were visualized using the FragPipe-PDV viewer.

### Statistical analysis

Statistical significance of differences between two groups was calculated by Welch's test (two-tailed, unpaired) using GraphPad Prism 8 (GraphPad Software). Details are provided in Table S4. *p* < 0.05 was considered statistically significant.

## Data availability

All supporting data are included in the main article and its Supporting information.

## Supporting information

This article contains supporting information.

## Conflicts of interest

The authors declare that they have no conflicts of interest with the contents of this article.

## References

[1] Ritz-Timme S., Collins M. (2002). Racemization of aspartic acid in human proteins. Ageing Res. Rev..

[2] Shapira R., Austin G.E., Mirra S.S. (1988). Neuritic Plaque amyloid in Alzheimer’s disease is highly racemized. J. Neurochem..

[3] Roher A.E., Lowenson J.D., Clarke S., Wolkow C., Wang R., Cotter R.J. (1993). Structural alterations in the peptide backbone of beta-amyloid core protein may account for its deposition and stability in Alzheimer’s disease. J. Biol. Chem..

[4] Masters P.M., Bada J.L., Zigler J.S. (1978). Aspartic acid racemization in heavy molecular weight crystallins and water insoluble protein from normal human lenses and cataracts. Proc. Natl. Acad. Sci. U. S. A..

[5] Sugiki T., Utsunomiya-Tate N. (2013). Site-specific aspartic acid isomerization regulates self-assembly and neurotoxicity of amyloid-β. Biochem. Biophys. Res. Commun..

[6] Tochio N., Murata T., Utsunomiya-Tate N. (2019). Effect of site-specific amino acid D-isomerization on β-sheet transition and fibril formation profiles of Tau microtubule-binding repeat peptides. Biochem. Biophys. Res. Commun..

[7] Murata T., Ito G., Utsunomiya-Tate N. (2023). Site-specific amino acid D-isomerization of Tau R2 and R3 peptides changes the fibril morphology, resulting in attenuation of Tau aggregation inhibitor potency. Biochem. Biophys. Res. Commun..

[8] Mcfadden P.N., Clarke S. (1982). Methylation at D-aspartyl residues in erythrocytes: possible step in the repair of aged membrane proteins. Proc. Natl. Acad. Sci. U. S. A..

[9] Kinouchi T., Ishiura S., Mabuchi Y., Urakami-Manaka Y., Nishio H., Nishiuchi Y. (2004). Mammalian d-aspartyl endopeptidase: a scavenger for noxious racemized proteins in aging. Biochem. Biophys. Res. Commun..

[10] Takahashi S., Ogasawara H., Hiwatashi K., Hori K., Hata K., Tachibana T. (2006). Paenidase, a novel d-aspartyl endopeptidase from Paenibacillus sp. B38: purification and substrate specificity. J. Biochem..

[11] Nirasawa S., Nakahara K., Takahashi S. (2018). Cloning and characterization of the novel d-aspartyl endopeptidase, paenidase, from Paenibacillus sp. B38. J. Biochem..

[12] Jumper J., Evans R., Pritzel A., Green T., Figurnov M., Ronneberger O. (2021). Highly accurate protein structure prediction with AlphaFold. Nature.

[13] Baek M., DiMaio F., Anishchenko I., Dauparas J., Ovchinnikov S., Lee G.R. (2021). Accurate prediction of protein structures and interactions using a three-track neural network. Science.

[14] Paysan-Lafosse T., Blum M., Chuguransky S., Grego T., Pinto B.L., Salazar G.A. (2023). InterPro in 2022. Nucleic Acids Res..

[15] Zhang M., Zhang L., Guo R., Xiao C., Yin J., Zhang S. (2022). Structural basis for the catalytic activity of filamentous human serine beta-lactamase-like protein LACTB. Structure.

[16] Bennett J.A., Steward L.R., Rudolph J., Voss A.P., Aydin H. (2022). The structure of the human LACTB filament reveals the mechanisms of assembly and membrane binding. PLoS Biol..

[17] Keckesova Z., Donaher J.L., De Cock J., Freinkman E., Lingrell S., Bachovchin D.A. (2017). LACTB is a tumour suppressor that modulates lipid metabolism and cell state. Nature.

[18] Zeng K., Chen X., Hu X., Liu X., Xu T., Sun H. (2018). LACTB, a novel epigenetic silenced tumor suppressor, inhibits colorectal cancer progression by attenuating MDM2-mediated p53 ubiquitination and degradation. Oncogene.

[19] Li H.-T., Dong D.-Y., Liu Q., Xu Y.-Q., Chen L. (2019). Overexpression of LACTB, a mitochondrial protein that inhibits proliferation and invasion in glioma cells. Oncol. Res. Featur. Preclin. Clin. Cancer Ther..

[20] Cutano V., Ferreira Mendes J.M., Escudeiro-Lopes S., Machado S., Vinaixa Forner J., Gonzales-Morena J.M. (2023). LACTB exerts tumor suppressor properties in epithelial ovarian cancer through regulation of Slug. Life Sci. Alliance..

[21] Zeng K., Huang N., Liu N., Deng X., Mu Y., Zhang X. (2024). LACTB suppresses liver cancer progression through regulation of ferroptosis. Redox Biol..

[22] Wingo A.P., Liu Y., Gerasimov E.S., Gockley J., Logsdon B.A., Duong D.M. (2021). Integrating human brain proteomes with genome-wide association data implicates new proteins in Alzheimer’s disease pathogenesis. Nat. Genet..

[23] Wang Y.H., Luo P.P., Geng A.Y., Li X., Liu T.-H., He Y.J. (2023). Identification of highly reliable risk genes for Alzheimer’s disease through joint-tissue integrative analysis. Front. Aging Neurosci..

[24] Masaki T., Nakamura K., Isono M., Soejima M. (1978). A new proteolytic enzyme from Achromobacter lyticus M497-1. Agric. Biol. Chem..

[25] Breddam K., Meldal M. (1992). Substrate preferences of glutamic-acid-specific endopeptidases assessed by synthetic Peptide Substrates based on intramolecular fluorescence quenching. Eur. J. Biochem..

[26] Kakudo S., Kikuchi N., Kitadokoro K., Fujiwara T., Nakamura E., Okamoto H. (1992). Purification, characterization, cloning, and expression of a glutamic acid-specific protease from Bacillus licheniformis ATCC 14580. J. Biol. Chem..

[27] Polianskyte Z., Peitsaro N., Dapkunas A., Liobikas J., Soliymani R., Lalowski M. (2009). LACTB is a filament-forming protein localized in mitochondria. Proc. Natl. Acad. Sci. U. S. A..

[28] Tunyasuvunakool K., Adler J., Wu Z., Green T., Zielinski M., Žídek A. (2021). Highly accurate protein structure prediction for the human proteome. Nature.

[29] Mirdita M., Schütze K., Moriwaki Y., Heo L., Ovchinnikov S., Steinegger M. (2022). ColabFold: making protein folding accessible to all. Nat. Methods.

[30] Holm L. (2022). Dali server: structural unification of protein families. Nucleic Acids Res..

[31] Meng E.C., Goddard T.D., Pettersen E.F., Couch G.S., Pearson Z.J., Morris J.H. (2023). UCSF ChimeraX : tools for structure building and analysis. Protein Sci..

[32] Ito G., Tomita T., Utsunomiya-Tate N. (2023). LRRK2-mediated phosphorylation and thermal stability of Rab12 are regulated by bound nucleotides. Biochem. Biophys. Res. Commun..

[33] Kong A.T., Leprevost F.V., Avtonomov D.M., Mellacheruvu D., Nesvizhskii A.I. (2017). MSFragger: ultrafast and comprehensive peptide identification in mass spectrometry–based proteomics. Nat. Methods.

