# Supplementary material for: Discovery and biochemical characterization of the D-aspartyl endopeptidase activity of the serine protease LACTB[image]
Source: J Biol Chem. 2025 Apr 24;301(6):108549. doi: 10.1016/j.jbc.2025.108549 (PMC12148432; doi:10.1016/j.jbc.2025.108549)
Supplement: Supporting information [file mmc1.docx]

# Supporting Information

**Discovery and biochemical characterization of the d-aspartyl endopeptidase activity of the serine protease LACTB**

Genta Ito and Naoko Utsunomiya-Tate

- Supplementary experimental procedures
- Figure S1
- Figure S2
- Figure S3
- Figure S4
- Figure S5
- Table S1
- Table S2
- Table S3
- Table S4
- Table S5

# Supplementary experimental procedures

## Plasmid construction

A vector backbone, pET-15b-6His-SUMO1 (DU51062), was a generous gift from Professor Dario Alessi (University of Dundee, UK). cDNA of hLACTB (UniProt ID: P83111) (97–547 aa), hLACTBL1 (UniProt ID: H0Y608) (63–450 aa), hGLSL (UniProt ID: Q9UI32) (148–478 aa) and hGLSK (UniProt ID: O94925) (221–533 aa) were codon-optimized for expression in *E. coli* and synthesized by Eurofins Genomics, Japan. The PCR primers used to subclone the open reading frames (ORFs) are listed in Table S1. The complete nucleotide sequences of the ORFs are listed in Table S2. Plasmids for bacterial expression encoding N-terminal 6His-SUMO1-fusion proteins were constructed by inserting the amplified ORFs into pET-15b-6His-SUMO1 using the HiFi assembly master mix (New England Biolabs) according to the manufacturer's instructions. The S164A mutation in hLACTB was introduced by site-directed mutagenesis using the following oligonucleotides as primers: 5'-ccgttatgcgtattgcggccatttcaaaaagcctg-3' and 5'-caggctttttgaaatggccgcaatacgcataacgg-3'. All plasmids used in this study and their full details are available from the corresponding author.

## Protein expression and purification

Protein expression in *E. coli* and purification using Ni-NTA agarose was performed as described previously (32), except for the buffer compositions. The sonication buffer was 50 mM Tris-HCl, 250 mM NaCl, 1 mM DTT, 1 mM PMSF, pH 8.0. The wash buffer was 50 mM Tris-HCl, 250 mM NaCl, 1 mM DTT, 20 mM imidazole, pH 8.0. The elution buffer was 50 mM Tris-HCl, 250 mM NaCl, 1 mM DTT, 500 mM imidazole, pH 8.0. After elution, the buffer was exchanged with storage buffer (20 mM Tris-HCl, 150 mM NaCl, 1 mM DTT, 10% (v/v) glycerol, pH 8.0) using PD-10 desalting columns (Cytiva). The protein concentration was determined by the Bradford assay on a plate reader (VersaMax or SpectraMax i3x; Molecular Devices). One milligram of Ni-eluates was further purified by SEC on an ÄKTA pure 25 instrument (Cytiva) using a Superdex 200 Increase 10/300 GL column (Cytiva) for 6His-SUMO1-hLACTB (97–547) and a Superdex 75 10/300 GL column (Cytiva) for 6His-SUMO1-hGLSL (148–478) and 6His-SUMO1-hGLSK (221–533). The SEC columns were pre-equilibrated with SEC buffer (20 mM Tris-HCl, 150 mM NaCl, 1 mM DTT, pH 8.0), and proteins were eluted in the same buffer. Fractions containing 6His-SUMO1-fusion proteins were pooled and dialyzed against the storage buffer. Purification was validated by running SDS-PAGE gels stained with Coomassie brilliant blue and scanned with a flat-bed scanner (Brother Industries, Japan). Purified proteins were aliquoted, snap-frozen in liquid nitrogen, and stored at −80 °C until use. The molecular weights of 6His-SUMO1-hLACTB (97–547), 6His-SUMO1-hGLSL (148–478), 6His-SUMO1-hGLSK (221–533) are 63.2, 48.5, 46.4 kDa, respectively.

## Validation of purified proteins by LC-MS/MS

One microgram of purified protein was diluted to 50 μL with 0.1%(w/v) RapiGest SF (Waters) dissolved in 50 mM ammonium bicarbonate. The protein solution was incubated at 60 °C for 30 min for denaturation. To reduce cysteine residues, Tris(2-carboxyethyl) phosphine (TCEP; Fujifilm Wako, Japan) was added to a final concentration of 5 mM, and the mixtures were incubated at 56 °C for 30 min. The mixtures were then supplemented with methyl methanethiosulfonate (#23011; Thermo Fisher Scientific) to a final concentration of 10 mM to alkylate the reduced cysteines and incubated at room temperature for 30 min in the dark. Mixtures were supplemented with 0.4 μg Mass Spec Grade Trypsin/Lys-C mix (Promega) and incubated overnight at 37 °C with shaking at 1000 rpm.

To precipitate RapiGest, the mixtures were added with trifluoroacetic acid (TFA; Fujifilm Wako) at a final concentration of 0.5%(v/v), shaken at 1000 rpm for 1 h at 37 °C, and centrifuged at 12,000 g for 10 min at room temperature. The supernatants were desalted using GL-Tip SDB (GL Sciences, Japan) and eluted in 70% TFA/0.1% formic acid (FA) (Kanto Kagaku, Japan). The solvent was evaporated using a vacuum evaporator (EZ-2; Scrum Inc., Japan), and the dried peptides were dissolved in 20 μL of 0.1% FA.

One microliter of the samples was injected into a nano-HPLC (Vanquish Neo; Thermo Fisher Scientific) and separated using a 5 μm C_18_ trap column (#174500; Thermo Fisher Scientific) and a 3 μm C_18_ analytical column (#360/75-3-12; Nikkyo Technos, Japan) at a constant flow rate of 300 nL/min with a gradient 0%B to 45%B for 10 min, using 0.1% FA and 0.1%FA/80% acetonitrile as solvent A and B, respectively. The eluted peptides were transferred to a Q Exactive Orbitrap mass spectrometer (MS) (Thermo Fisher Scientific) using a Nanospray Flex ion source (Thermo Fisher Scientific). The MS was operated in positive mode with the following parameters. For the full scan, lock masses: 391.28429 and 445.12003 (use if all present); sensitivity: 70,000; AGC target: 3×10^6^; maximum ion integration time (IT): 60 msec; scan range: 380-1500 m/z. For data-dependent MS2 (Top 10); sensitivity: 17,500; AGC target: 1×10^5^; maximum IT: 55 msec; isolation window: 1.6 m/z; scan range: 200-2000 m/z; normalized collision energy (NCE): 27. For data-dependent acquisition; minimum AGC target: 4.55×10^2^; intensity threshold: 8.3×10^3^; charge exclusion: unassigned, 1, and >5; dynamic exclusion: 5 s.

Raw mass spectrometry data were searched with FragPipe v22.0 against a combined protein database downloaded from UniProt, consisting of the reference proteomes of *Homo sapiens* (UP000005640) and *Escherichia coli* (strain K12) (UP000000625). Amino acid sequences of mature paenidase was added to the database to exclude possible contamination of purified proteins with paenidase. Reverse sequences of all entries were added as decoys and used to control the false discovery rate (FDR). Common contaminants (<https://www.thegpm.org/crap/>) were also added. An FDR of 0.01 was set for both protein and peptide identification. The parameters used in MSFragger were as follows: Precursor mass tolerance: -5 and 5 ppm; Fragment mass tolerance: 0.02 Da; Protein digestion: "stricttrypsin" (missed cleavages: up to 2); Variable modifications: Methionine oxidation (+15.9949); Fixed modifications: Cysteine alkylation by MMTS (+45.98772). Proteins without unique peptide assignment are excluded. These experiments were performed once, and the raw and result files are deposited at the ProteomeXchange Consortium via jPOSTrepo with the identifier PXD062910 and are provided as Table S5.

# Figure S1


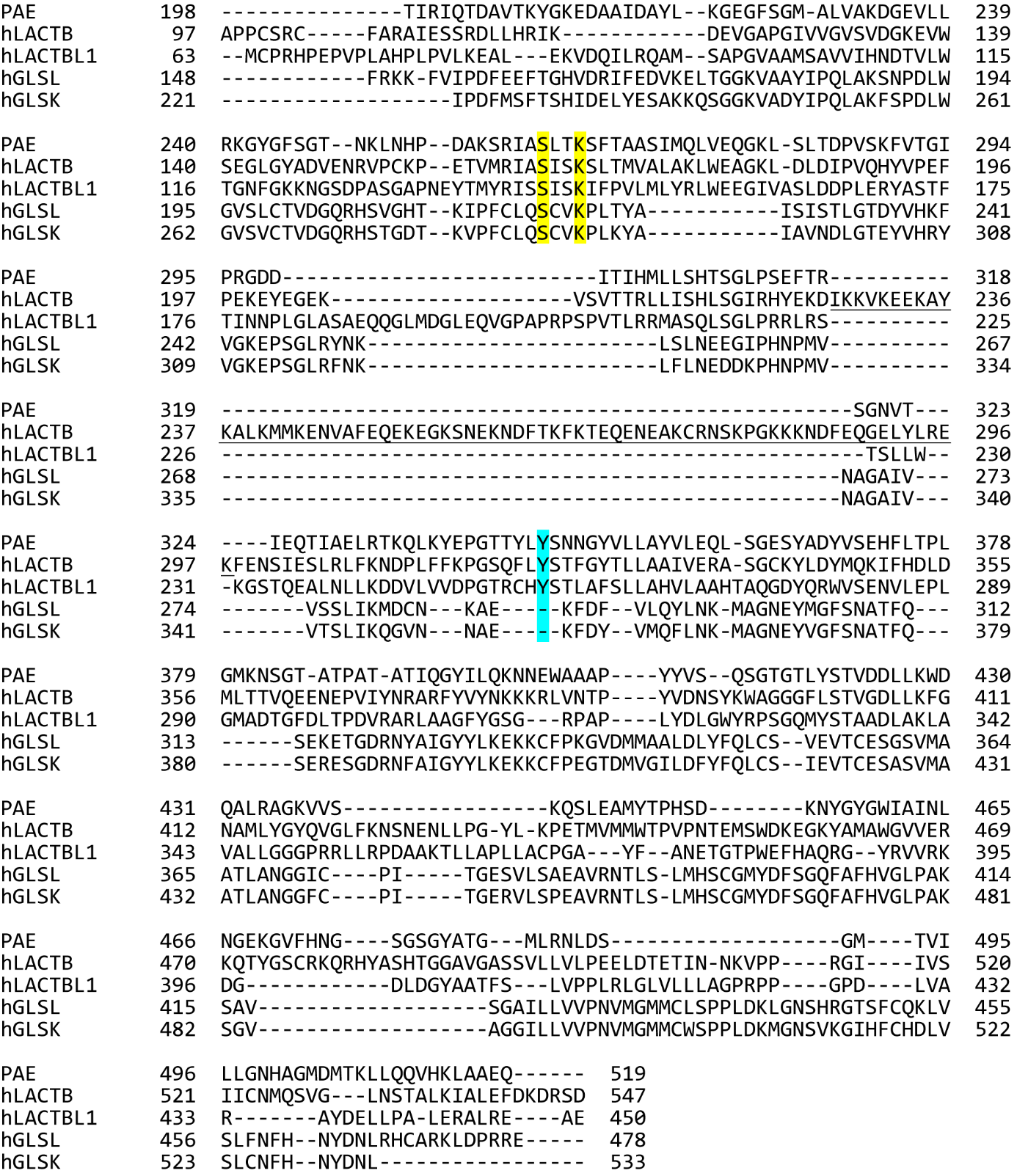


An alignment of amino acid sequences of mature paenidase (PAE; 198–519 aa) (Uniprot #A0A2Z6BCG6), human LACTB (hLACTB; 97–547 aa) (Uniprot #P83111), human LACTBL1 (hLACTBL1; 63–450 aa) (Uniprot #H0Y608), human GLSL (hGLSL; 148–478 aa) (Uniprot #Q9UI32), and human GLSK (hGLSK; 221–533 aa) (Uniprot #O94925). Alignment was performed using the Clustal Omega algorithm on SnapGene version 5.1.7 (GSL Biotech). Catalytic residues (Ser262 and Lys265) of paenidase that are conserved among beta-lactamases are highlighted in yellow. Catalytic Tyr346 of paenidase conserved in hLACTB and hLACTBL1 but not in hGLSL and hGLSK is highlighted in cyan. The region not determined in the cryo-EM structure (dashed line in Figure 1C) in hLACTB is underlined.

# Figure S2


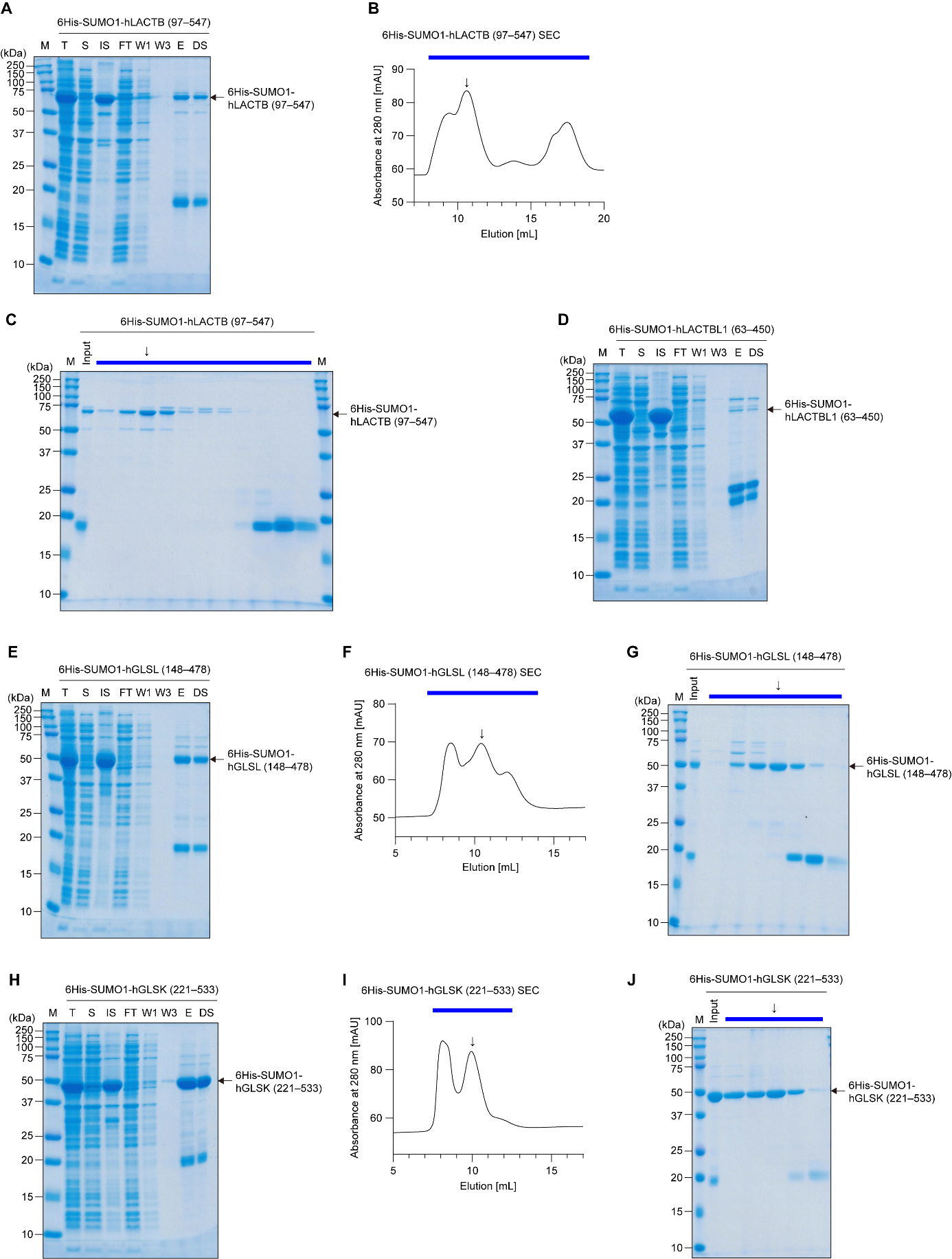


Purification of 6His-SUMO1-hLACTB (97–547), hLACTBL1 (63–450), hGLSL (148–478), hGLSK (221–533). (A, D, E, H) Proteins expressed in *E. coli* were purified by His-tag affinity chromatography. M, molecular weight marker; T, total lysate; S, soluble fraction; IS, insoluble fraction; FT, flow-through; W1, first wash; W3, third wash; E, eluate; DS, desalted sample. Size exclusion chromatography (SEC) purification of 6His-SUMO1-hLACTB (97–547) (B, C), 6His-SUMO1-hGLSL (148–478) (F, G) and hGLSK (221–533) (I, J). Bands corresponding to the proteins of interest are marked with arrows on the side of the gels, and the SEC fractions marked with arrows on the top of the gels were used for assays.

# Figure S3


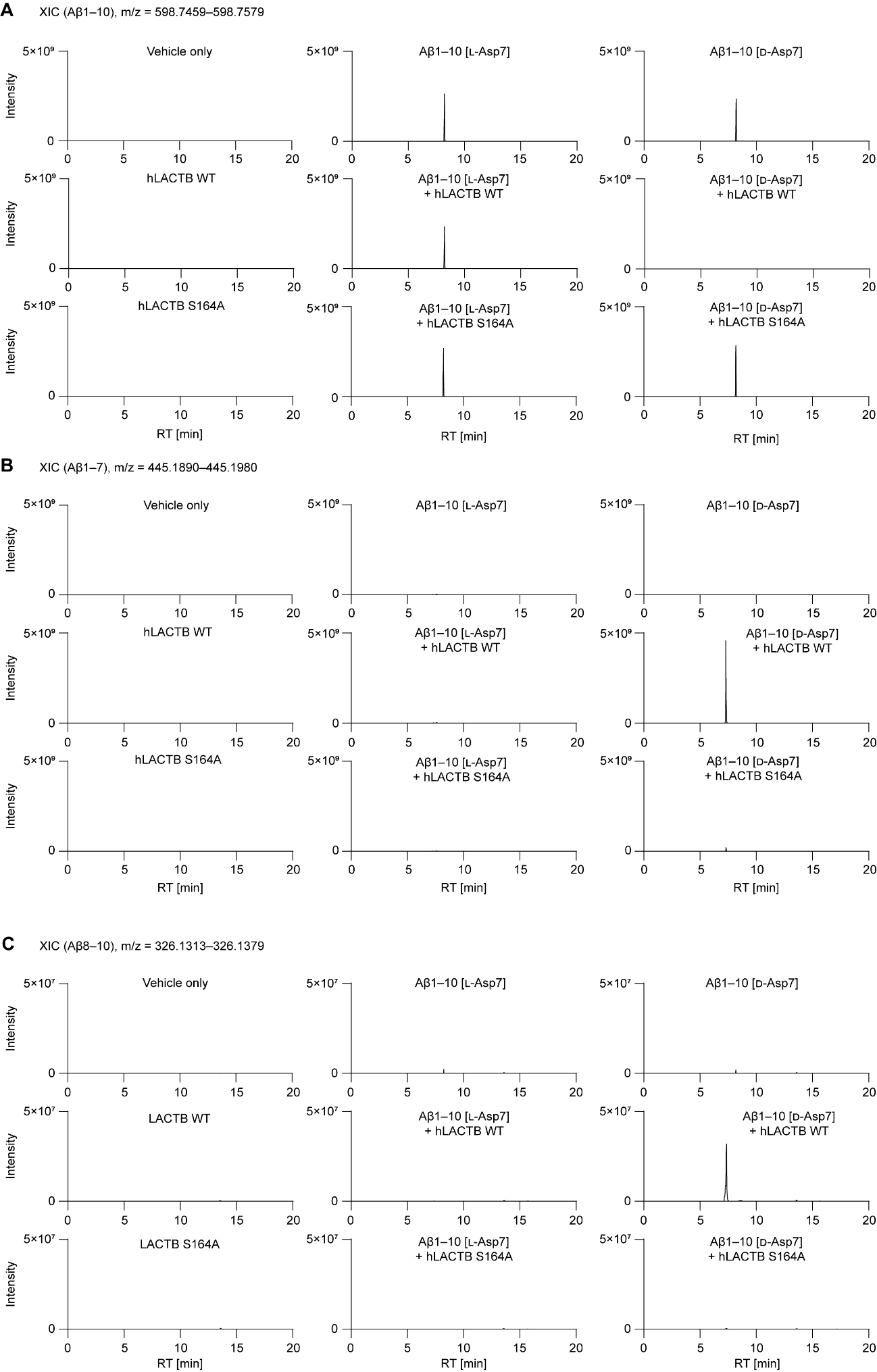


Extracted ion chromatograms (XICs) of precursor ions corresponding to Aβ1–10, Aβ1–7 and Aβ8–10. (A) XICs of a precursor ion of m/z 598.7459–598.7579 corresponding to Aβ1–10 (NH_2_-DAEFRHDSGY-COOH) with charge +2. (B) XICs of a precursor ion of m/z 445.1890–445.1980 corresponding to Aβ1–7 (NH_2_-DAEFRHD-COOH) with charge +2. (C) XICs of a precursor ion of m/z 326.1313–326.1379 corresponding to Aβ8–10 (NH_2_-SGY-COOH) with charge +1.

# Figure S4


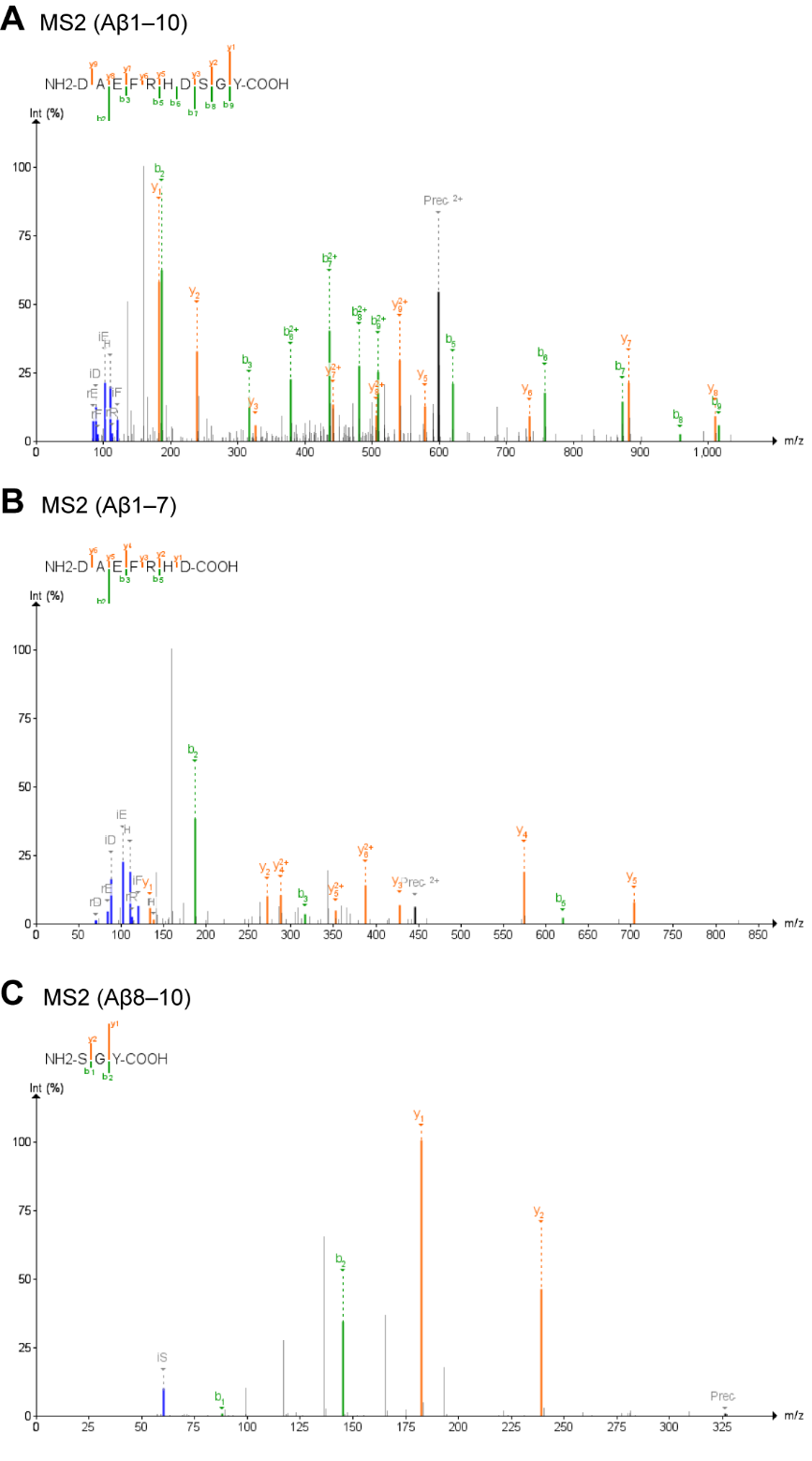


Representative MS2 spectra of Aβ1–10, Aβ1–7 and Aβ8–10. (A) A representative MS2 spectrum of a precursor ion of m/z 598.7518 (RT = 8.138 min) detected in the Aβ1–10 [d-Asp7] sample without 6His-SUMO1-hLACTB (97–547) (upper right panel in Figure S3A). (B, C) Representative MS2 spectra of precursor ions of (B) m/z 445.1929 (RT = 7.441 min) and (C) m/z 326.1345 (RT = 7.190 min) detected in the Aβ1–10 [d-Asp7] sample with 6His-SUMO1-hLACTB (97–547) WT (middle right panel in Figure S3B and S3C, respectively).

# Figure S5


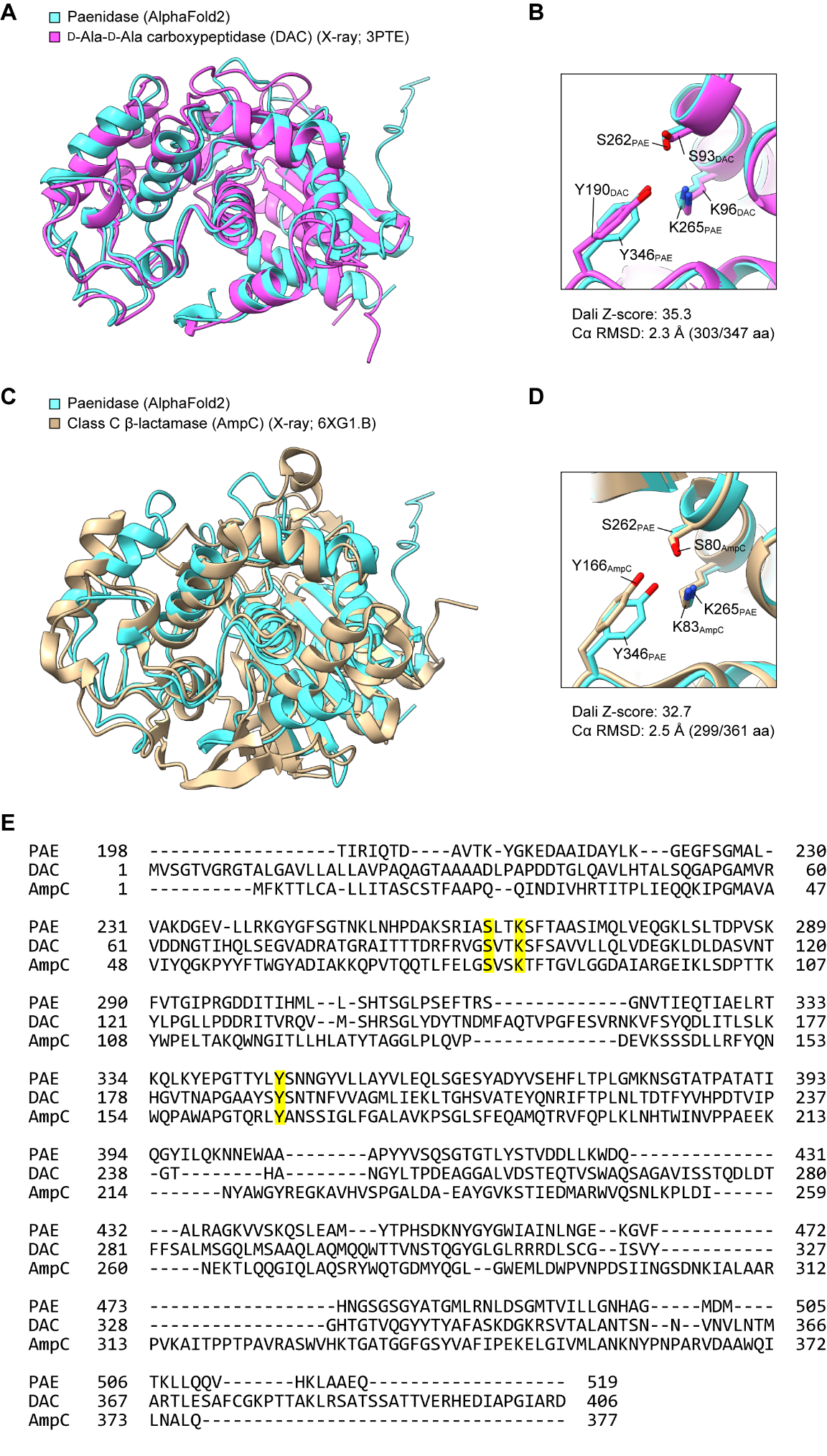


Structural comparison between paenidase and other bacterial beta-lactamase-like proteins. (A) Superposition of the structure of paenidase predicted by AlphaFold2 (cyan) and D-Ala-D-Ala carboxypeptidase (DAC) from *Streptomyces* sp. R61 determined by X-ray crystallography (magenta) (PDB ID: 3PTE). (C) Superposition of the structure of paenidase predicted by AlphaFold2 (cyan) and class C β-lactamase (AmpC) from *E. coli* determined by X-ray crystallography (pale brown) (PDB ID: 6XG1, chain B). (B, D) Close-up of the superimposed putative catalytic residues, with oxygen and nitrogen atoms marked in red and blue, respectively. The Dali Z-score and Cα RMSD are shown below the panel. (E) An alignment of amino acid sequences of mature paenidase (PAE; 198–519 aa) (Uniprot #A0A2Z6BCG6), DAC from *Streptomyces* sp. R61 (Uniprot #P15555), and AmpC from *E.coli* (Uniprot #P00811). Catalytic residues (Ser262, Lys265, Tyr346) of paenidase that are conserved among β-lactamase-like proteins and shown in (B, D) are highlighted in yellow.

# Table S1

Nucleotide sequences of primers used for subcloning of ORFs into the pET-15b-6His-SUMO1 vector.

| ORF | Nucleotide sequence (5′ → 3′) |
| --- | --- |
| hLACTB (97–547) | aggaacaaacggggggggccccaccgtgttctcg |
|  | gccggatctttacgcttaatccgagcgatctttatcgaattccag |
| hLACTBL1 (63–450) | aggaacaaacgggggggatgtgcccgcgtcatccc |
|  | ccggatctttacgcttattcagcttcacgcagtgc |
| hGLSL (148–478) | aggaacaaacggggggctttcgcaagaaattcgttatcccggat |
|  | gccggatctttacgcttattcgcgacgtggatcgagt |
| hGLSK (221–533) | aggaacaaacggggggcatcccggattttatgagctttactagc |
|  | gccggatctttacgcttacagattgtcatagttgtggaagttgcaaag |

# Table S2

Complete nucleotide sequences of the ORFs of (A) hLACTB (97–547), (B) hLACTBL1 (63–450), (C) hGLSL (148–478), and (D) hGLSK (221–533).

(A)

| hLACTB (97–547) |
| --- |
| GCCCCACCGTGTTCTCGGTGCTTTGCGCGAGCCATTGAATCGTCGCGCGATCTGCTTCATCGCATCAAAGATGAAGTTGGTGCACCGGGCATTGTCGTCGGTGTTAGTGTTGATGGCAAAGAGGTCTGGTCTGAAGGTTTAGGTTATGCGGATGTGGAAAACCGTGTTCCATGCAAACCCGAAACCGTTATGCGTATTGCGAGCATTTCAAAAAGCCTGACCATGGTAGCACTTGCCAAACTCTGGGAAGCTGGCAAACTGGATTTGGACATACCGGTCCAACACTATGTACCGGAATTTCCGGAGAAAGAGTATGAAGGCGAAAAAGTGAGCGTGACAACGCGTCTGTTGATCAGTCATCTGTCTGGCATTCGTCACTACGAGAAAGACATCAAAAAGGTGAAAGAAGAAAAAGCCTACAAAGCGTTAAAGATGATGAAAGAGAATGTTGCATTCGAACAGGAAAAAGAAGGTAAGTCCAATGAGAAAAACGACTTTACCAAATTCAAAACCGAACAGGAGAATGAAGCCAAATGTCGCAATAGCAAACCTGGCAAAAAGAAGAACGACTTTGAACAAGGCGAACTGTATTTACGCGAAAAGTTTGAAAACAGCATTGAAAGCCTTCGGCTGTTCAAAAATGATCCGCTGTTTTTCAAACCTGGTAGTCAGTTCCTGTATTCCACGTTTGGCTATACCTTACTGGCTGCGATTGTCGAACGTGCTTCAGGTTGCAAGTACCTCGATTACATGCAGAAAATCTTTCACGACCTGGATATGCTCACTACAGTTCAGGAAGAAAACGAGCCGGTGATCTATAATCGTGCACGTTTCTACGTGTACAACAAAAAGAAACGCCTGGTTAACACTCCGTATGTGGACAACAGCTACAAATGGGCTGGAGGGGGCTTTCTGTCGACCGTAGGGGATTTACTCAAATTTGGGAATGCCATGTTGTATGGGTATCAGGTGGGACTGTTCAAGAACTCCAACGAGAACTTGCTGCCAGGGTACCTAAAACCGGAAACCATGGTGATGATGTGGACGCCTGTACCGAATACCGAGATGAGCTGGGATAAGGAGGGCAAATATGCGATGGCATGGGGTGTAGTGGAACGTAAACAGACGTATGGCTCATGTCGCAAACAACGCCATTACGCGTCACATACAGGTGGTGCGGTTGGAGCAAGTAGTGTCCTGCTGGTGCTTCCGGAGGAACTAGACACCGAGACGATTAATAACAAGGTCCCCCCAAGAGGCATTATTGTGTCCATCATCTGCAACATGCAATCTGTGGGCCTGAATTCGACTGCCTTGAAAATCGCGCTGGAATTCGATAAAGATCGCTCGGAT |

(B)

| hLACTBL1 (63–450) |
| --- |
| ATGTGCCCGCGTCATCCCGAACCGGTACCGCTGGCTCATCCGTTACCGGTACTGAAAGAAGCGTTGGAGAAAGTTGACCAGATTCTGCGCCAAGCCATGAGCGCACCTGGTGTGGCGGCCATGTCTGCCGTCGTCATTCACAACGATACCGTGTTATGGACGGGCAACTTCGGGAAGAAGAATGGCTCGGACCCAGCCTCTGGAGCCCCAAACGAATACACGATGTATCGCATTTCCTCGATCAGCAAAATCTTTCCGGTGCTGATGTTGTATCGCCTGTGGGAAGAGGGTATTGTGGCCAGTCTTGATGATCCGCTCGAACGCTATGCGTCAACGTTTACCATCAACAATCCCCTGGGCCTGGCTAGCGCTGAACAGCAGGGGTTAATGGATGGGCTGGAACAAGTGGGCCCAGCCCCACGTCCTAGTCCCGTGACTCTCCGCCGGATGGCATCCCAGTTGTCAGGCCTGCCTCGCCGGCTTCGCTCGACGAGCCTGCTGTGGAAAGGCTCAACCCAGGAAGCTCTGAACTTACTCAAAGATGACGTGCTTGTTGTCGATCCGGGTACACGGTGCCACTACTCGACATTGGCGTTCAGCCTGTTGGCCCATGTTCTGGCGGCCCATACCGCTCAGGGTGACTATCAGCGTTGGGTGAGCGAGAATGTCCTGGAACCCCTGGGCATGGCGGATACCGGCTTTGACCTGACTCCGGATGTTCGCGCGCGTTTGGCAGCAGGCTTCTATGGGAGTGGTCGTCCTGCACCGCTGTACGATCTGGGCTGGTATCGTCCGTCTGGGCAAATGTACAGTACCGCAGCGGACTTAGCGAAACTGGCGGTAGCACTGCTGGGCGGTGGTCCACGCCGCTTGCTGCGTCCAGATGCCGCGAAAACCCTGTTAGCGCCACTTCTCGCATGTCCGGGAGCGTACTTTGCCAATGAAACGGGTACCCCGTGGGAGTTTCACGCTCAACGTGGTTATCGCGTTGTGCGCAAAGACGGCGATCTGGATGGCTATGCCGCGACTTTCTCCCTCGTCCCGCCGTTACGCCTGGGTCTGGTACTGCTTCTTGCGGGTCCGCGTCCTCCGGGACCGGACCTGGTTGCACGCGCGTACGATGAGCTCCTGCCTGCCTTAGAGCGTGCACTGCGTGAAGCTGAA |

(C)

| hGLSL (148–478) |
| --- |
| TTTCGCAAGAAATTCGTTATCCCGGATTTTGAAGAGTTTACTGGACATGTTGATCGCATCTTTGAAGATGTGAAAGAGTTAACCGGCGGTAAAGTCGCAGCGTATATTCCGCAACTCGCCAAATCGAACCCGGATCTGTGGGGTGTTTCGTTGTGCACGGTTGACGGTCAACGCCATTCCGTTGGGCACACGAAAATCCCCTTCTGCCTGCAGAGTTGTGTCAAACCACTGACGTATGCGATTAGTATCAGCACACTGGGGACGGACTATGTGCACAAATTCGTCGGGAAAGAACCTAGCGGTTTGCGCTATAACAAACTGTCTCTGAATGAAGAAGGCATTCCGCATAATCCGATGGTGAATGCTGGTGCCATTGTGGTGTCATCTCTCATCAAGATGGATTGCAACAAAGCGGAGAAATTCGACTTTGTGCTGCAGTACCTGAACAAGATGGCGGGCAATGAATACATGGGTTTCAGCAACGCTACCTTTCAGTCCGAGAAAGAAACCGGAGATCGGAACTATGCGATTGGCTATTACTTGAAAGAGAAGAAGTGCTTTCCAAAAGGCGTAGACATGATGGCCGCACTTGATCTGTACTTTCAGTTATGCAGTGTTGAAGTGACCTGTGAAAGCGGAAGTGTGATGGCAGCCACTCTTGCGAATGGCGGCATTTGCCCCATTACCGGTGAAAGCGTACTGTCTGCAGAAGCAGTACGTAACACCCTGAGCCTGATGCATTCGTGTGGCATGTATGACTTCTCCGGGCAATTTGCGTTTCATGTCGGTTTACCGGCCAAATCTGCCGTTTCAGGCGCGATTCTGTTGGTAGTCCCGAATGTGATGGGCATGATGTGCCTTTCACCGCCTCTGGATAAACTGGGCAATTCCCATCGTGGTACAAGCTTCTGTCAGAAACTGGTGTCGCTGTTCAACTTTCACAATTACGACAACTTACGCCACTGTGCTCGTAAACTCGATCCACGTCGCGAA |

(D)

| hGLSK (221–533) |
| --- |
| ATCCCGGATTTTATGAGCTTTACTAGCCACATCGATGAACTGTACGAAAGTGCCAAGAAACAGAGCGGTGGGAAAGTTGCCGACTATATCCCTCAGTTGGCGAAATTTTCACCCGATTTATGGGGTGTGTCGGTCTGTACGGTTGATGGCCAACGGCATTCTACAGGTGATACCAAAGTACCGTTCTGTCTCCAGTCATGCGTGAAACCGCTCAAGTATGCAATTGCCGTGAACGATCTGGGAACGGAGTATGTGCATCGCTATGTTGGCAAAGAGCCTTCGGGTTTACGCTTTAACAAACTGTTCCTTAACGAAGATGACAAGCCCCATAATCCGATGGTGAATGCAGGCGCTATTGTGGTTACCTCTCTGATCAAACAAGGCGTCAATAACGCCGAGAAATTCGACTATGTAATGCAGTTTCTGAACAAAATGGCCGGCAATGAGTATGTCGGATTTTCGAACGCGACCTTTCAGAGCGAACGTGAAAGTGGCGATCGTAACTTTGCGATTGGTTACTACCTGAAAGAGAAGAAATGCTTCCCGGAAGGAACGGATATGGTCGGGATTCTGGACTTCTACTTTCAGCTGTGCTCCATCGAAGTCACCTGTGAATCAGCTTCGGTTATGGCAGCTACACTGGCGAATGGCGGGTTTTGTCCGATTACTGGCGAACGTGTGTTGAGTCCAGAAGCGGTACGCAATACCCTGTCTCTCATGCATTCCTGTGGCATGTACGATTTCAGCGGTCAATTCGCGTTTCACGTAGGTTTACCGGCGAAAAGCGGTGTTGCAGGCGGTATTTTGCTTGTTGTCCCGAATGTGATGGGTATGATGTGCTGGAGTCCACCACTGGATAAAATGGGGAACAGCGTGAAAGGCATTCACTTCTGCCATGACCTGGTGTCCCTTTGCAACTTCCACAACTATGACAATCTG |

# Table S3

Parameters for LC-MS/MS analysis of Aβ1-10 and peptides generated by LACTB.

| LC parameters | |
| --- | --- |
| Apparatus | Vanquish Neo (Thermo Fisher Scientific) |
| Columns | Trap column: 5 μm C_18_ trap column (#174500; Thermo Fisher Scientific)  Analytical column: 3 μm C_18_ analytical column (#360/75-3-12; Nikkyo Technos, Japan) |
| Solvents | A: 0.1% formic acid  B: 80% acetonitrile / 0.1% formic acid |
| Flow rate | 300 nL/min |
| Gradient | 0–45%B for 10 min |

| MS parameters | |
| --- | --- |
| Apparatus | Q Exactive (Thermo Fisher Scientific) |
| Ion source | Nanospray Flex (Thermo Fisher Scientific) |
| Mode | Positive |
| *Full scan* | |
| Lock masses | 391.28429, 445.12003 (use if all present) |
| Resolution | 70,000 |
| AGC target | 3×10^6^ |
| Max IT | 60 ms |
| Scan range | 200–2000 m/z |
| *Data dependent MS2 (Top 5)* | |
| Resolution | 17,500 |
| AGC target | 1×10^5^ |
| Max IT | 55 ms |
| Isolation window | 1.6 m/z |
| Scan range | 200–2000 m/z |
| Normalized collision energy | 27 |
| *Data dependent acquisition* | |
| Min AGC target | 4.55×10^2^ |
| Intensity threshold | 8.3×10^3^ |
| Charge exclusion | unassigned, >5 |
| Dynamic exclusion | 1 s |

# Table S4

A summary of the *P*-values and the statistical tests used in the figures.

| **Fig.** | **Comparison** | **n** | **Test** | ***P*-value** |
| --- | --- | --- | --- | --- |
| 2C | WT vs. S164A | 3 | Normality: Shapiro-Wilk test  Welch's t-test (two-tailed; unpaired) | 0.00150 |
| 2D | D-Asp vs. L-Asp | 3 | Normality: Shapiro-Wilk test  Welch's t-test (two-tailed; unpaired) | 0.00017 |

# Table S5 (separate Excel file)

The complete list of proteins observed in the purified fractions of LACTB WT and S164A. Proteins with no unique peptides were excluded. Raw data have been deposited in jPOSTrepo, a member repository of the ProteomeXchange consortium, under the identifier PXD062910. Complete lists of peptides identified in the LACTB WT and S164A samples are shown in separate tabs.
